# Massive transient pulmonary air embolism during permanent cardiac pacemaker implantation

**DOI:** 10.21542/gcsp.2018.16

**Published:** 2018-06-30

**Authors:** Emre Ozdemir, Fatma Kayaalti Esin, Cem Nazli

**Affiliations:** Katip Celebi University, Atatürk Training and Research Hospital, Cardiology Clinic, Izmir, Turkey

## Abstract

Pulmonary air embolism is a rare complication with a high probability of death. We present an air embolism case during permanent cardiac pacemaker implantation procedure. When the patient worsened hemodynamically, we saw a large air embolism in the main pulmonary trunk. Air embolism can be fatal, it is always iatrogenic, but is an avoidable complication.

## Case study

**Figure 1.**
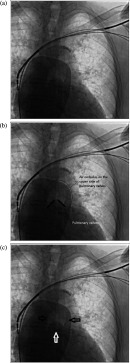
a) Air embolus on the upper side of pulmonary valve. b) Air embolus noted on the pulmonary valve in the circle. c) Black arrows indicating air embolus, white arrow showing pulmonary valve.

Pulmonary air embolism is a complication whereby gas enters into heart cavities via the venous entrance to pulmonary artery. The implantation of a pacemaker may cause several complications^1^. The most common complications are bleeding and infection - but other complications can include cardiac and/or vascular injuries. Air embolism is a rare but potentially lethal complication.

A 68-year-old patient with 25% LVEF was admitted and ICD was planned. After subclavien vein puncture, an 8F peel-away sheath (without the haemostatic valve) was inserted. When sheath dilatator was removed, a rumbling-like sound was heard. Both O_2_ saturation and arterial tension of the patient started to decrease.

**Figure 2.**
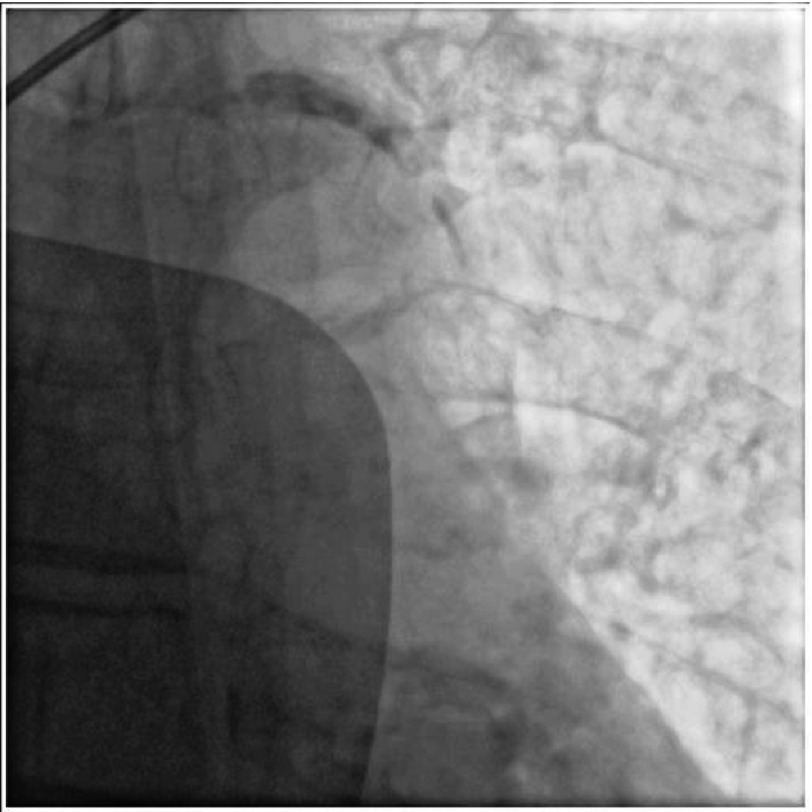
Air embolus disappeared spontaneously with airway support, high flow oxygen, vasopressors treatment.

Via thoracoscopy we saw a large air embolism in the main pulmonary trunk, instead of pneumothorax (Figure 1). Treatment with airway support, high flow oxygen, and vasopressors was started immediately. 6F sheath was inserted to femoral vein for aspiration of embolus via a JR4 guiding catheter, but before the intervention, symptoms spontaneously improved; TA and vascular oxygen saturation had increased and we saw that most of the air embolus had disappeared (Figure 2).

To minimise air embolism during lead insertion, it is recognised that it is important to press the neck of the sheath once stylet is removed, until the introduction of the lead^2^, but the best way to avoid air embolism is to use a sheath with haemostatic valve. It is also important to note that air embolism can cause a cerebral embolism if there is a veno-arterial shunt, such as an atrial septal defect.
